# Cosmetic Eyebrow Tattoo Removal Using a Q-Switched Ruby Laser: A Case Report

**DOI:** 10.7759/cureus.103215

**Published:** 2026-02-08

**Authors:** Luis L Velázquez Arenas, Sarahi Garay Enriquez, Daniela Gómez Guerra

**Affiliations:** 1 Dermatology, School of Medicine and Health Sciences at Tecnológico de Monterrey, Monterrey, MEX; 2 Medicine, School of Medicine and Health Sciences at Tecnológico de Monterrey, Monterrey, MEX

**Keywords:** adverse effects, cosmetic tattoo, eyebrow tattoo removal, qs ruby laser, tattoo removal

## Abstract

Tattoos are created by the deposition of exogenous pigment into the dermis and were historically considered permanent; however, advances in laser technology have made their removal increasingly feasible. Q-switched (QS) laser systems remain the standard of care for tattoo removal, operating through selective photothermolysis. Despite their effectiveness, laser tattoo removal may be associated with transient or persistent adverse effects. Here, we report the case of a 38-year-old woman who presented with a bilateral cosmetic eyebrow tattoo exhibiting blue-green discoloration and an unnatural appearance, refractory to seven prior laser treatments performed elsewhere. The patient was treated with five sessions of a QS 694 nm ruby laser between March and August 2024, resulting in progressive pigment lightening. Transient erythema and edema were observed, as well as a reduction in eyebrow hair density, which was managed with adjunctive topical therapy containing 5% minoxidil, tretinoin, phytantriol, and vitamin E. This case highlights the effectiveness of the QS ruby laser for eyebrow tattoo removal and emphasizes eyebrow hair density reduction as a potential adverse effect that should be addressed during patient counseling and informed consent.

## Introduction

Tattoos are created by the deposition of exogenous pigment into the dermal layer of the skin, resulting in long-lasting alterations in skin coloration. Although tattoos were historically considered permanent, advances in laser technology over recent decades have made partial or complete removal achievable through a variety of therapeutic modalities. As a result, tattoo removal has become a commonly performed procedure in dermatologic practice [1].

Current approaches to tattoo removal are broadly categorized into ablative and non-ablative techniques. Non-ablative Q-switched (QS) laser systems remain the standard of care, operating on the principle of selective photothermolysis to fragment tattoo pigment while minimizing damage to surrounding tissue. Treatment efficacy is influenced by multiple factors, including pigment composition, tattoo depth, and skin phototype, making appropriate wavelength selection essential [2]. However, laser tattoo removal may be associated with transient local reactions such as erythema, edema, blistering, crusting, and pain, as well as residual tattoo outlines or textural changes; permanent adverse effects may also occur, including scarring, hyperpigmentation, or hypopigmentation, and pigment color alteration [3]. Here, we present a case of cosmetic eyebrow tattoo removal using a QS ruby laser, demonstrating pigment clearance with a favorable cosmetic outcome. The patient experienced transient local reactions, including edema and erythema, and an uncommon finding of reduced eyebrow hair density, which was treated with topical minoxidil but did not fully recover.

## Case presentation

**Figure 1:**
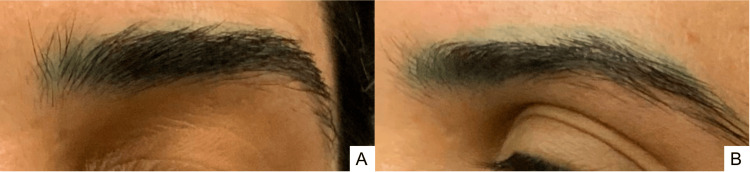
Baseline clinical photographs Frontal (A) and lateral (B) views of the blue-green discoloration of the cosmetic eyebrow tattoo before laser treatment

A 38-year-old female presented to our clinic seeking removal of a bilateral cosmetic eyebrow tattoo. She reported having undergone seven previous laser sessions at an outside facility using unspecified laser technologies, without achieving noticeable clinical improvement. Upon examination, she presented an unnatural look of the eyebrows with blue-green discoloration (Figures 1A, 1B). Given the pigment coloration, treatment with a QS 694 nm ruby laser (Discovery Pico®) was selected. All sessions were performed with topical anesthetic cream applied under occlusion with plastic wrap one hour before each session, followed by epidermal cooling using ice immediately before laser application. Additionally, post-treatment care included application of Cicaplast®, a re-epithelializing agent containing panthenol, for one week.

A total of five treatment sessions using QS 694 nm ruby laser at a pulse frequency of 1 Hz were performed between March and August 2024, at 4-week intervals. Treatment began at a fluence of 3.6 J/cm². In the second session, a 3-mm square spot size was used, with fluence increased to 5 J/cm² along the superior external perimeter of the eyebrows and 4 J/cm² within the eyebrow body. During the third session, the same spot size was maintained, and fluence was set at 5 J/cm², resulting in a pronounced frosting response consistent with effective pigment photofragmentation. In the fourth and fifth sessions, fluence was further increased to 5.5 J/cm², while maintaining a consistent spot size. Progressive clinical lightening of the tattoo pigment was observed throughout treatment.

**Figure 2:**
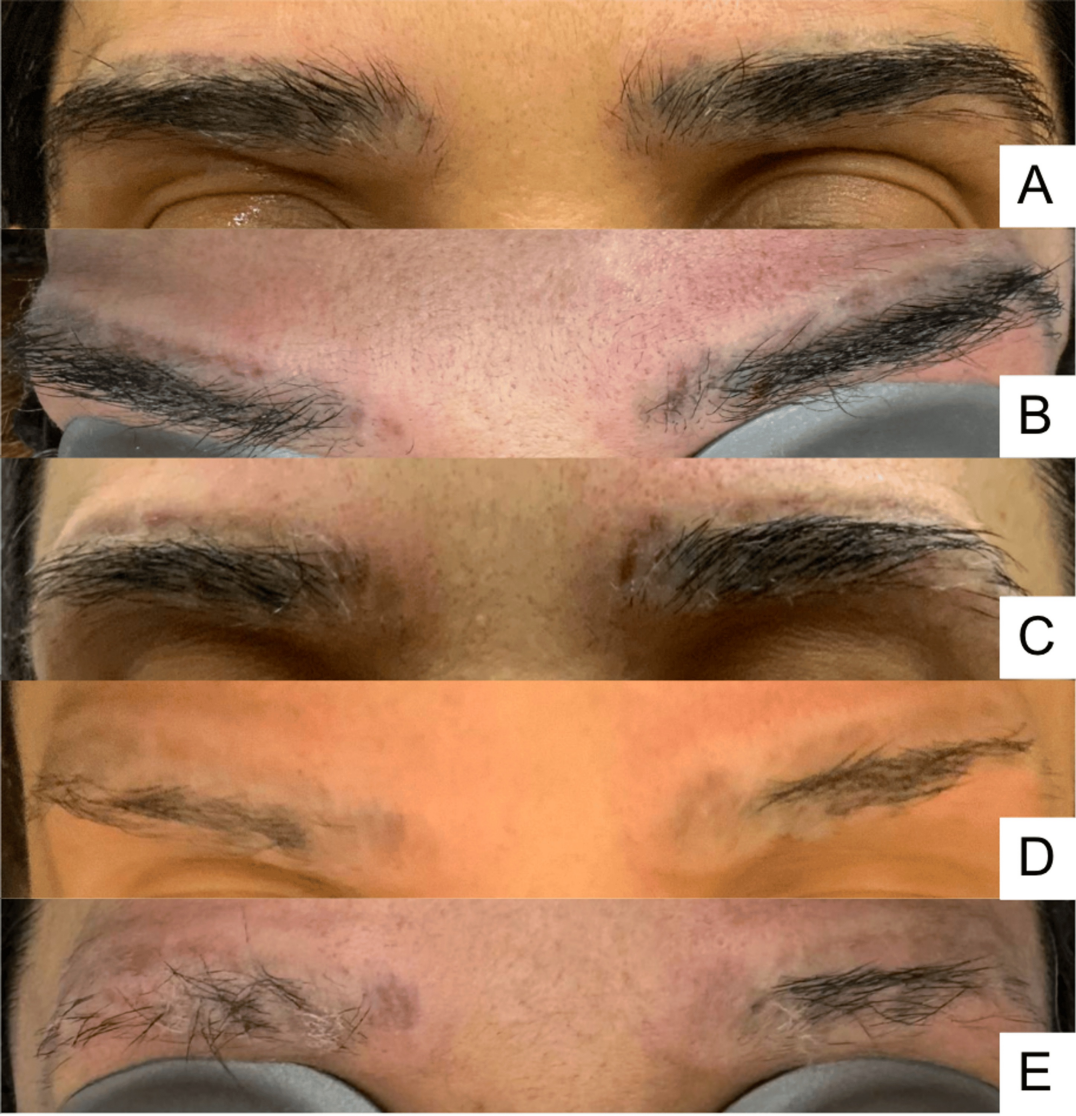
Serial clinical photographs obtained after each laser session (A) After the first session (March 14, 2024), (B) after the second session (April 22, 2024), (C) after the third session (May 21, 2024), (D) after the fourth session (July 6, 2024), and (E) after the fifth and final session (August 6, 2024)

**Figure 3:**
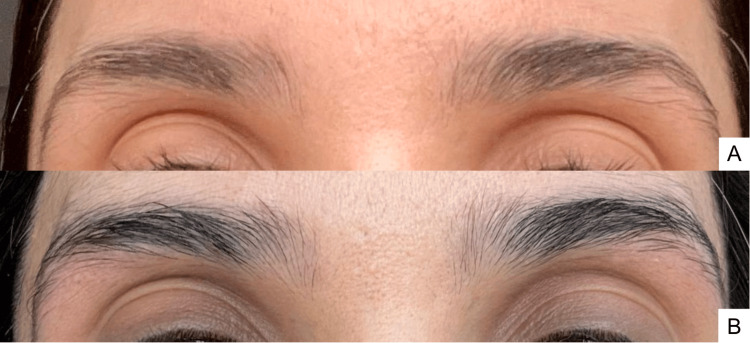
Follow-up clinical photographs Images obtained at seven months after completion of laser treatment while receiving topical 5% minoxidil therapy (A) and at 1 year and 3 months follow-up (B)

Serial clinical photographs demonstrated substantial and uniform fading of the blue-green pigment, with complete clearance achieved by the conclusion of laser therapy (Figures 2A-2E). During and after treatment, the patient developed transient erythema and mild edema, as well as an uncommon reduction in eyebrow hair density. Following tattoo clearance, the patient initiated adjunctive therapy aimed at promoting eyebrow regrowth with topical hair lotion containing 5% minoxidil, 0.025% tretinoin, 0.2% phytantriol, and 1% vitamin E, which was continued for a total duration of one year and three months. At the end of this period, sustained pigment clearance and satisfactory cosmetic outcomes were confirmed, with preservation of normal skin texture and color (Figures 3A, 3B).

## Discussion

QS laser systems deliver high-energy pulses of very short duration, confining the photoacoustic effect to pigment-containing particles while minimizing injury to surrounding tissue. This process results in fragmentation of tattoo ink, facilitating subsequent clearance through dermal macrophage phagocytosis and lymphatic transport [4]. According to Kuperman-Beade et al., the QS ruby laser (694 nm) demonstrates high absorption by melanin and blue-black and green tattoo pigments, supporting its use in the present case [5]. Adverse reactions following QS laser treatment have been reported in approximately one quarter of treated patients, with hypopigmentation being the most frequently described adverse effect [4,5]. Scarring has also been described following laser tattoo removal, with certain anatomic locations, such as the neck and shoulders, appearing to be at higher risk [4]. When scarring involves the eyebrow region with associated hair loss, reconstructive options such as a scalp strip grafting technique have been reported as effective corrective approaches [6]. In our patient, a reduction in eyebrow hair density was observed; however, this finding was not consistent with permanent scarring, as partial hair regrowth was achieved with topical minoxidil therapy.

## Conclusions

This case highlights the effectiveness of the QS ruby laser for cosmetic eyebrow tattoo removal while underscoring the importance of recognizing potential adverse effects beyond those commonly reported. In this context, a reduction in eyebrow hair density may occur and should be considered a possible adverse effect or complication. Awareness of this outcome may assist in patient counseling and informed consent.
